# Pig productive performance parameters and costs in Spain: evolution from 2015 to 2024

**DOI:** 10.1186/s40813-026-00500-w

**Published:** 2026-03-05

**Authors:** Josep Font, Joan Rocadembosch, Josep Bernaus, Olga Miró, Judit Argerich, Laia Cordón, Irene Planas, Just Font, Lorenzo Fraile

**Affiliations:** 1 SIP Consultors SL, Prats del Lluçanes, Spain; 2 Departament de Ciéncia Animal, ETSEA, University de Lleida, Lleida, Spain; 3 AgrotecnioCenter, Lleida, Spain

**Keywords:** Cost, Performance parameters, Swine, Temporal trend

## Abstract

**Background:**

The goals of the present work were to describe reliable reference values and their temporal evolution for production parameters and pig production cost from 2015 to 2024 in Spain. Between 109 and 123 pig production companies from Spain were included in this study from 2015 to 2024. Monthly data on feed consumption, number of pigs produced, expenses and census collected in herds of these companies were standardized and used to calculate cost and production parameters. The collected data each month was merged to obtain a yearly average value considering the pig production flow each month. A generalized linear mixed model was performed to evaluate the association between the production performance and cost variables and the year, geographical location and its interaction.

**Results:**

The production performance has been continuously improving in the piglet production and fattening phase from 2015 to 2020 and from 2015 to 2022, respectively. Thus, the number of piglets per sow per year increased 0.4 by year and the feed conversion ratio decreased 0.025 by year during the former time periods. This trend changed suddenly in 2020 and 2022 for the piglet production and fattening phase, respectively. The introduction of a high virulent PRRS strain (HP-PRRSV) may have played a key role. On the other hand, the production performance in the nursery phase has been continuously worsening since 2015 probably due to the reduction of antibiotic use since this year, the ban of zinc oxide in 2022 and the introduction of the HP-PRRSV since 2020. On the other hand, feed price was quite constant from 2015 to 2020 but suffered a huge increment between 2021 and 2022 and decreased afterwards. The total cost per kilogram of body weight produced showed a similar pattern to the one observed for feed price. This result highlights the relevance of the feed price in the final cost and the impact of health events despite continuous improvement in production performance by genetic selection, management, facilities and feed quality.

**Conclusion:**

Pig production parameters have generally improved from 2015 to 2020 and worsened afterwards. The cost of pig production has been significantly affected by the feed cost and by the production parameters, especially sow productivity and post-weaning mortality.

**Supplementary Information:**

The online version contains supplementary material available at 10.1186/s40813-026-00500-w.

## Background

Pig production plays a key role for global meat supplies representing 34% of the world´s meat consumption [1]. Since the 1990s, the European Union has experienced a steady increase in pork production that has been limited in the last years due to the many challenges faced by this industry [2]. Health challenges caused by African Swine Fever in several European countries [3] plus the presence of high virulence PRRSV strains in countries like Italy [4] and Spain [5] are key factors affecting the European pig sector. Another key factor is the shortage of personnel to work in pig farms due to many social factors such as the aging farm population, the low density of population in rural areas, the difficulties to achieve a generational renewal and the lack of social recognition of this profession [6–9]. Finally, stricter regulations on antimicrobial use [10], welfare and environmental impact are also three key factors contributing to the slowdown in pork production growth in Europe [2].

The evolution of the pig population in the European Union has been uneven between countries; Spain increased its global pig census by 35.1% from 2013 to 2023 whereas other key pig producers like Germany, France, Denmark, The Netherlands and Poland decreased their census approximately by 25, 12, 8, 13 and 11%, respectively in the same period [11]. One explanation for this divergent evolution in Spain may be its role in the global pig market due to its great potential to export to Europe and Asian countries probably related to companies with a vertically integrated production system and direct access to big retailers [12]. All in all, Spain is the first pig producer in Europe and one of the most important in the world. Moreover, the pig production sector is crucial to the Spanish economy accounting for 17.2% and 42.6% of the agricultural and livestock production respectively [13]. Thus, it is critical to maintain competitiveness to hold this position in the future at worldwide level.

The knowledge of pig production cost is necessary for rational decision-making based on cost-benefit analysis at the producer level. Moreover, the relative weight of each production parameters on the final cost of pig production must be determined to decrease the cost as much as possible and increase competitiveness. Performance indicators commonly employed to assess swine productivity include total piglets weaned per sow per year. This parameter can be calculated analysing the number of piglets weaned per sow per litter and the number of litters produced per sow per year. In the same way, the number of piglets weaned per sow per litter is calculated from the number of piglets born alive per sow and the preweaning mortality. Finally, to correctly calculate the piglet cost at weaning, the annual feed consumption per sow must be known. This parameter combined with the sow productivity allows calculating the feed consumption per weaned piglet [14, 15].

During the nursery and fattening, average daily gain (ADG) and feed conversion ratio (FCR) are key performance indicators [16]. Mortality also plays an important role, as elevated rates among growing pigs are associated with reduced profitability in swine production systems [17]. These metrics are therefore used to quantify the impact of diseases during the rearing period [18]. Reliable performance indicators are key to measure accurately the effects that disease has within the swine industry. This calculation is the basis for estimating the cost-benefit of each control and prevention strategy to optimise resource allocation [19–21]. Currently, there is a paucity of real and updated data regarding pig production parameters and associated costs that could serve as industry references, reflecting the intrinsic challenges of collecting information. The objectives of the present study were: (1) to produce reliable reference values for production performance parameters and pig production costs for Spain between 2015 and 2024; and (2) to describe their trends over time.

## Material and methods

### Pig production companies included in this study

From 2015 to 2024, a total of 109 to 123 Spanish pig production companies were included in the study. These enterprises operated farrow-to-finish, two-site, and three-site production systems. In all cases, piglets born within the same week were grouped and managed as a cohort. Weaning occurred between three and four weeks of age, after which, pigs entered the nursery phase until reaching an average body weight of approximately 19 kg. Then, pigs were transferred to finishing facilities, where they remained until achieving an average slaughter weight that has progressively increased during the study period (from 107 kg in 2015 to 119 Kg in 2024). At this point, pigs were sent to slaughterhouses for processing. Participating companies were geographically distributed across Spain and grouped in three different geographical areas (east, north and south) considering factors such as climate and the distribution of the pig population across the country. Thus, east (Catalonia, Aragon and Navarra), north (Galicia and Castilla León) and south zone (Comunidad valenciana, Andalucia, Murcia and Castilla la Mancha) represents approximately 69%, 13% and 18%, respectively according to our database in the study period. The combined sow inventory represented in the study ranged from 553.561 in 2015 to 1.211.319 in 2024 that means 22.6% and 46.4% of the sow total inventory at national level, respectively.

Each month, companies submitted data on feed consumption, production, operating expenses, and herd census to a specialized consultancy (SIP Consultors SL, Spain- https://www.sipconsultors.com/en/), which conducts economic analyses of swine production systems across various European countries. The collected data—Summarized in Table 1 for each production phase—were standardized by SIP Consultors SL to ensure comparability between companies by year. For the purposes of this study, the final weights for the weaning, nursery, and finishing phases were standardized to 6 kg, 19 kg, and 107, 108, 109, 110, 111, 113, 114, 113, 116 and 119 Kg from 2015 to 2024, respectively. Monthly records were aggregated to produce annual average values, considering the monthly production flow. Finally, average daily gain and feed conversion ratio during the fattening and total production period were also standardized to 113 kilograms for each year, to avoid any bias in comparisons due to the slaughterhouse weight change across the study period.

**Table 1:** Monthly collected data from each pig production company

Production phase	Variable
Gestation and lactation	Number of piglets born alive per litter (NBA)
	Pre-weaning mortality, % (PM1)
	Number of piglets weaned per sow per litter (NW)
	Number of piglets produced per sow per year (NPWY)
	Number of cycles per sow per year (NCS)
	Feed price per sow, Euros/tonne (FP1)
	Kilograms of sow feed per weaned piglet (KFWP)
	Total kilograms of sow feed per year (TSF)
	Cost per weaned piglet, Euros (CWP1)
Nursery, from weaning to 19 kg of body weight	Nursery average daily gain, g/day (ADG2)
	Nursery feed conversion ratio (FCR2)
	Nursery mortality,% (NM2)
	Feed price for nursery, Euros/tonne (FP2)
	Cost per nursery piglet, Euros (CNP2)
Fattening, from 19 to final kg of body weight depending on the year (see material and methods section)	Fattening average daily gain, g/day (ADG3)
	Fattening feed conversion ratio (FCR3)
	Fattening mortality,% (FM3)
	Feed price for fattening, Euros/tonne (FP3)
	Total cost per pig, Euros (TCP3)
Whole production phase	Total feed conversion ratio (FCRT)
	Total cost per produced Kg of body weight (TCK)
	Total feed cost per pig, Euros (TFC)
	Total drug and vaccine cost per pig, Euros (DVCT)
	Total fixed cost per pig, Euros (TFIXC)
	Total reproduction cost per pig, Euros (TREPC)

## Statistical analyses

All statistical analyses were carried out using the SAS system V.9.1.3 (SAS institute Inc, Cary, NC, USA). Shapiro Wilk´s and Levene tests were used to evaluate the normality of the distribution of the variables and the homogeneity of variances, respectively. As most of the variables were non-normally distributed, descriptive statistics included median and interquartile range that was calculated for each variable during the period from 2015 to 2024. Each pig company was used as the experimental unit for further analysis. A generalized linear mixed model, using a normal distribution with link identity function and unbounded variance components, was performed to evaluate the association between the production performance and cost variables and the year (2015 to 2024) and geographical location (east, north and south) as fixed effects and the company as a random variable as this data was recorded each year (repeated measures). Moreover, an interaction between year and geographical area was also included in the model. The significance level (p) was set at 0.05 with statistical tendencies reported when *p* < 0.10.

## Results

### Piglet production phase

**Fig. 1:**
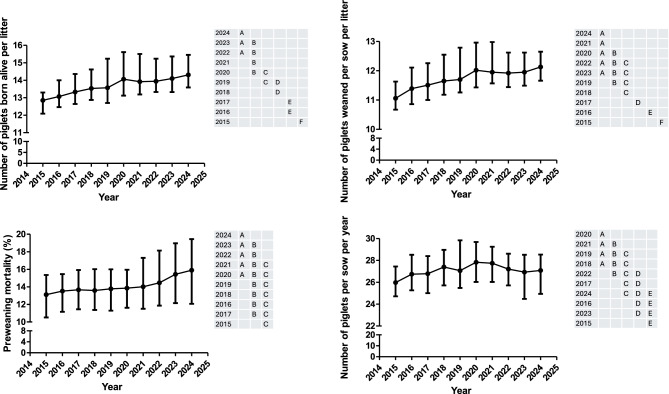
Evolution over time (median and interquartile range) of the number of piglets born alive per litter, percentage of pre-weaning mortality, number of piglets weaned per sow per litter and the number of piglets produced per sow per year during the piglet production phase. Years (rows) in the tables not sharing any letter were different (*p* < 0.05)

**Fig. 2:**
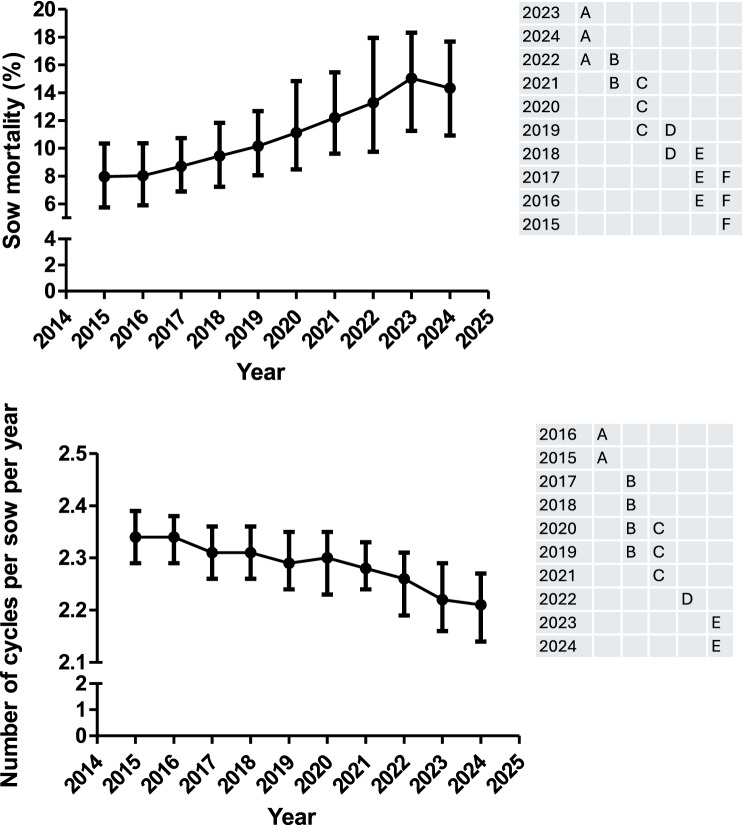
Evolution over time (median and interquartile range) of the number of cycles per sow per year and the percentage of sow mortality during the piglet production phase. Years (rows) in the tables not sharing any letter were different (*p* < 0.05)

The number of piglets born alive per litter (NBA), the number of piglets weaned per sow per litter (NW) and per year (NPWY) and the pre-weaning mortality (PM1) are shown in Fig. 1. The sow mortality and the number of cycles per sow and year (NCS) are shown in Fig. 2. Moreover, all the information for the former variables is available in supplementary table 1. The NBA, sow mortality and PM1 were associated with the year and the geographical zone with NBA and PM1 lower in the south than in east area whereas sow mortality was higher in the east than in the other geographical areas. There is no significant interaction between year and geographical area. On the other hand, NW, NPWY and NCS were only associated with year.

NBA, NW and NPWY have been increasing every year from 2015 to 2020. Regarding NBA and NW, no change has been shown during the 2020–2023 period, with only a slight improvement in 2024. The tendency to increase NPWY suddenly changed in 2021 where the number of NPWY started decreasing until 2023. In 2024, there was a slight uprise of the NPWY but reaching values like those observed in 2016. At the same time, the pre-weaning mortality (PM1) has steadily increased across the period 2015–2020 with a steep increase from 2020 to 2024. On the other hand, sow mortality and NCS has also been increasing and decreasing, respectively from 2015 to 2023.

**Fig. 3:**
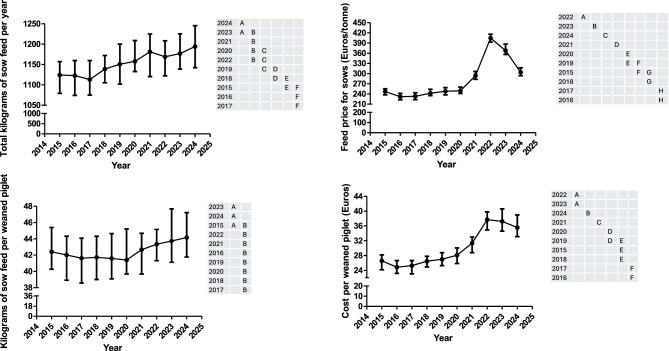
Evolution over time (median and interquartile range) of the feed price for sows, the total kilograms of sow feed per year, the kilograms of sow feed per weaned piglet and the cost per weaned piglet. Years (rows) in the tables not sharing any letter were different (*p* < 0.05)

The feed price per sow (FP1), the total kilograms of sow feed per year (TSF), the cost per weaned piglet (CWP1) and the kilograms of sow feed per weaned piglet (KFWP) are shown in Fig. 3. Moreover, all the information for the former variables is available in Supplementary Table 1. The FP1 and CWP1 were only associated with the year. On the other hand, KFWP and TSF were associated with the year and geographical area where a higher value was observed in the south than in the north and east in both cases. FP1 was quite constant from 2015 to 2020 but suffered a sharp increase between 2021 and 2022 and an important decrease until 2024. TSF has been steadily increasing during the study period, with slight changes in this tendency in years 2017 and 2022. On the other hand, KFWP have been regularly reduced from 2015 to 2017. In this year, the tendency for this parameter changed and a continuous increase was observed during the rest of the study period (except for 2020), with a steep slope between 2020 and 2024. Finally, the pattern observed for CWP1 is like the one described for KFWP.

### Nursery phase

**Fig. 4:**
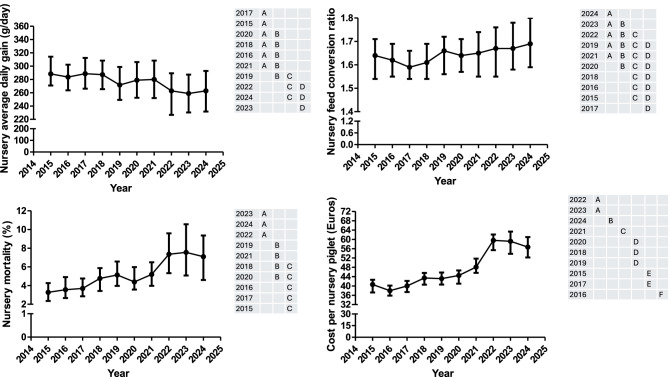
Evolution over time (median and interquartile range) of the average daily gain, the feed conversion ratio, the mortality and the cost per piglet during the nursery phase. Years (rows) in the tables not sharing any letter were different (*p* < 0.05)

The nursery average daily gain (ADG2), the nursery feed conversion ratio (FCR2), the nursery mortality (NM2), the cost per nursery piglet (CNP2) and the feed price for nursery (FP2) are shown in Fig. 4 and Supplementary Figure 1. Moreover, all the information for the former variables is available in Supplementary Table 2. The FP2 were only associated with the year. On the other hand, ADG2, FCR2, NM2 and CNP2 were associated with the year and geographical area. Thus, a higher value for ADG2 was observed in the south and north than the east whereas a higher value for FCR2 was observed in the south than in the other geographical areas. On the other hand, a higher value for NM2 and CNP2 was observed in the east than the north. There is no significant interaction between year and geographical area.

ADG2 showed a tendency to decrease from 2015 to 2024, whereas FCR2 increased, during the same period. Some differences have been observed for both parameters during this period, with an increase of the for ADG2 in 2017 and a decrease in FCR2 in 2020. NM2 has been increasing every year from 2015 to 2023 with slight fluctuations in 2017 and 2020. FP2 has been quite constant from 2015 to 2020, suffering a sharp increase between 2021 and 2022. Afterwards it has strongly decreased until 2024. At the end, CNP2 has only decreased from 2015 to 2016 and, afterwards, it has steadily increased until 2023 where a change of tendency is observed.

### Fattening production phase

**Fig. 5:**
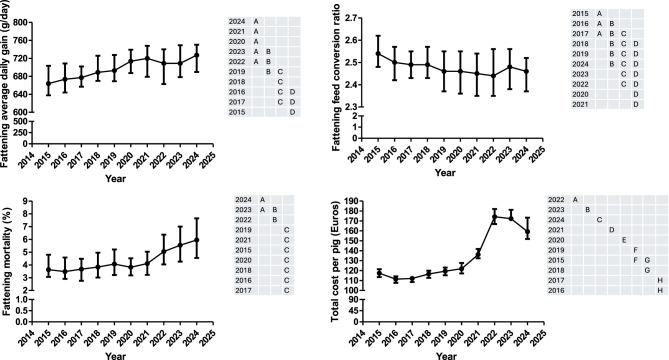
Evolution over time (median and interquartile range) of the average daily gain, the feed conversion ratio, the mortality and the cost per piglet during the fattening phase. Years (rows) in the tables not sharing any letter were different (*p* < 0.05)

The fattening average daily gain (ADG3), the fattening feed conversion ratio (FCR3), the fattening mortality (NM3), the total cost per pig (TCP3) and the feed price for fattening (FP3) are shown in Fig. 5 and Supplementary Figure 1. Moreover, all the information for the former variables is available in supplementary table 3. The NM3, TCP3, and FP3 were only associated with the year. On the other hand, FCR3 and ADG3 was associated with the year and geographical area. Thus, animals reared in the south showed a higher FCR3 than the value observed in the other geographical areas (north and east) whereas ADG3 was higher in the north than in the east and south. There is no significant interaction between year and geographical area.

A steadily increase was observed in the ADG3 from 2015 to 2024 except for the period 2021–2023 where these values do not change significantly. However, the pattern observed for ADG3 changed when this parameter was normalized to 113 Kg across the study period (Supplementary Figure 2) because it was observed a sharp decrease between 2021 and 2023 that was not observed without weight normalization (Fig. 5). FCR3 has been regularly decreasing from 2015 to 2021. In this year, this tendency changed and an increase in this parameter was observed until 2023. This pattern was very similar when FCR3 was normalized to 113 Kg across the study period (supplementary figure 2). NM3 has been increasing every year from 2015 to 2023 with slight fluctuations in the year 2017 and 2020 and a steep slope since 2021. Finally, FP3 showed a similar pattern to the sow and nursery feed (Fig. 3 and Supplementary Figure 1). At the end, TCP3 has only decreased from 2015 to 2016 and, afterwards, it has steadily increased until 2023 where a change of tendency is observed.

### Total production phase

**Fig. 6:**
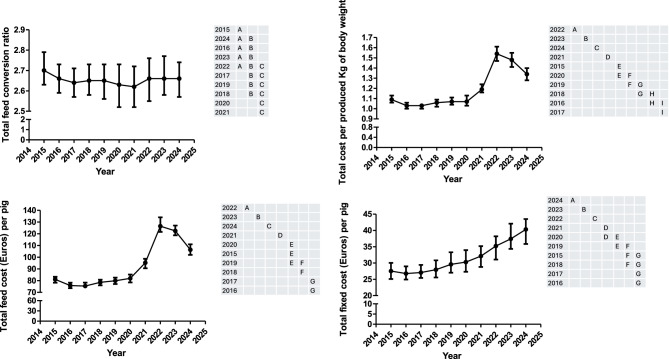
Evolution over time (median and interquartile range) of the total feed conversion ratio, total cost per produced kilogram of body weight, the total feed cost (Euros) per pig, and the total fixed cost (Euros) per pig. Years (rows) in the tables not sharing any letter were different (*p* < 0.05)

**Fig. 7:**
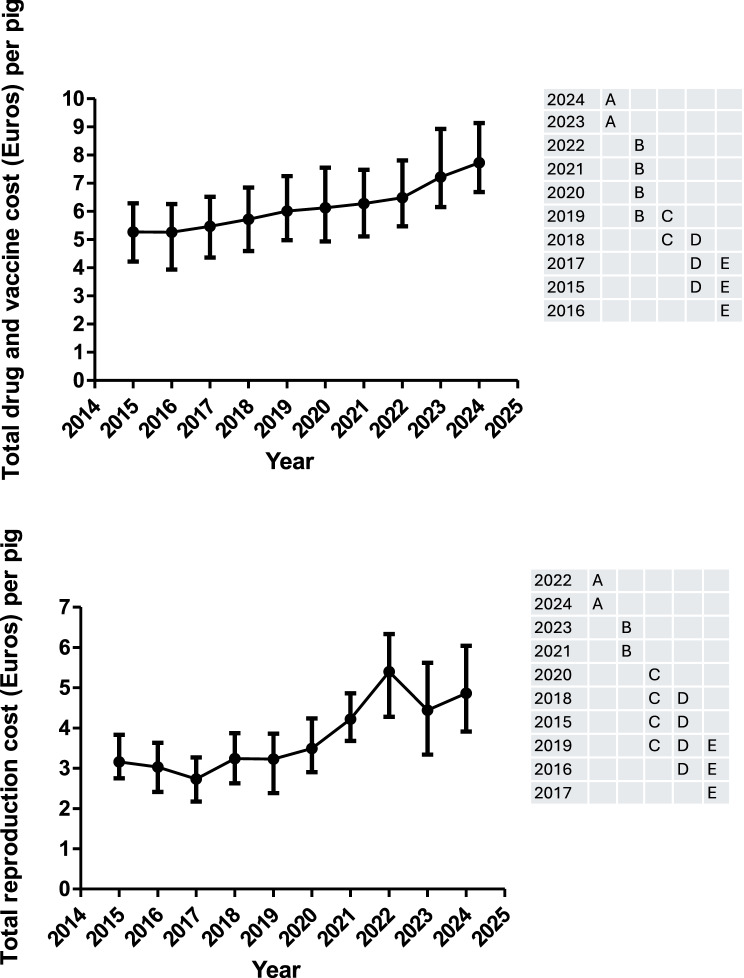
Evolution over time (median and interquartile range) of the total drug and vaccine cost (Euros) per pig and the total reproduction cost (Euros) per pig. Years (rows) in the tables not sharing any letter were different (*p* < 0.05)

The total feed conversion ratio (FCRT), the total cost per produced kg of body weight (TCK), the total feed cost per pig (TFC), the total drug and vaccine cost per pig (DVCT), the total fixed cost per pig (TFIXC) and the total reproduction cost per pig (TREPC) are shown in Figs. 6, 7 and Supplementary Figure 2. Moreover, all the information for the former variables is available in Supplementary Table 4. The TCK, TFIXC and TREPC were only associated with the year. On the other hand, FCRT and TFC was associated with the year and geographical area. Thus, animals reared in the south showed a higher FCRT and TFC than the value observed in the other geographical areas (north and east). There is no significant interaction between year and geographical area.

FCRT has been steadily decreasing from 2015 to 2021 and steadily increasing afterwards with a change of tendency in 2024. This pattern was very similar when FCRT was normalized to 113 Kg across the study period (Supplementary Figure 2). TFC and TCK decreased from 2015 to 2016 and steadily increased from 2017 to 2020. Afterwards, both parameters suffered a tremendous increase, reaching the maximum value in 2022. After that year, both decreased but not returned to the values observed in the previous years (2015–2020) (Fig. 6). The pattern observed for TFIXC and DCVT showed a steadily increase during the whole study period with a steep slope since 2021. However, the pattern observed for TREPC was like the previous ones until 2022, but it decreased afterwards.

## Discussion

According to official data, the pig census distribution in Spain [13] is 52%, 18%, and 23% for the East (Catalonia, Aragon, and Navarra), North (Galicia and Castilla y León), and South zones (Comunidad Valenciana, Andalucía, Murcia, and Castilla-La Mancha), respectively. These values are like the geographical distribution of the included companies in our database (69%, 13%, and 18% for East, North, and South zones, respectively). Thus, there is no substantial mismatch between the geographical distribution in this study and the national distribution of the pig population. Moreover, the combined sow inventory represented in the study ranged from 22.6% to 46.4% of the total national sow inventory over the study period. All in all, the reference values provided in this study are expected to be very close to the actual values for the entire pig population in Spain during the study period.

## Production parameters

A rise in the NBA piglets has been accompanied by corresponding increases in both the NW and the NPWY in the period 2010–2020 in Spain [22, this study]. Similar patterns have been reported in other European countries during this timeframe; however, NPWY in Spain consistently remained lower than the values observed in other European pig-producing countries such as Denmark, France, Germany, and the Netherlands [23]. The gradual increase in NPWY likely reflects significant advancements in breeding programs aimed at enhancing prolificacy in recent years [24]. Based on our findings in the period 2010–2020 [22, this study], NPWY in Spain was projected to rise by 0.4 piglets per year. However, this tendency to increase NPWY every year since 2010, changed suddenly in 2021 where a decrease in NPWY was observed from 28 in 2020 (maximum value observed in the study period) to 26.7 in 2023. This decrease in NPWY could be mostly explained a sharp decrease in NCS and an increase in PM1 from 2020 to 2024 that had been observed since 2015 but with a lower slope. This finding disagrees with the evolution observed for this key production parameter in other main European pig producing countries, where a steady increase is still observed [23]. One possible explanation for this finding might be partially due to a health event. A highly virulent strain of PRRSV-1, later named Rosalía [25], emerged in northeastern Spain in 2020 and rapidly spread nationwide. A peer-reviewed study [5] examined the consequences of this highly virulent PRRSV strain in a Spanish pig production system previously exposed to classical PRRSV strains. The study found that the new strain spread rapidly and caused severe productivity and health disruptions, comparable to the effects observed in PRRSV-naïve farms. This led to increased abortion rates, higher sow and piglet mortality, and more than 20% mortality in weaners and growing pigs [5, 26]. These observational findings have been corroborated under experimental conditions by our research group, confirming the threat posed by this new PRRSV strain to the Spanish pig production sector [27].

Productive performance indicators commonly employed include ADG and FCR [28]. During the period 2010–2014, both parameters have demonstrated improvement during the fattening phase, whereas remaining relatively unchanged during the nursery phase [22]. However, this pattern significantly changed in the period 2015–2024. Thus, both parameters have worsened in the nursery phase during this period being even worst between 2021 and 2023. Many factors could be involved in the outcome observed during the nursery period but the decrease in the use of antibiotics for fulfilling the European recommendations [10], the banning of the oxide zinc to control postweaning diarrhoea [29], and the presence of high virulence PRRSV strains in the Spanish pig population since 2020 could be key factors to be considered [5, 25]. It is well known that the nursery period is clinically affected with an impairment in productive parameters after a PRRSV outbreak in the sow population [30]. This situation was probably worsened due to the restrictions in the use of antibiotics to control bacterial diseases plus the ban to use zinc oxide to prevent postweaning diarrhoea. Both preventive medicine and other preventive tools have been highly effective for controlling nursery diseases; however, their use is no longer permitted in Europe due to international recommendations on the prudent use of antimicrobials and the ban on pharmacological doses of zinc oxide. Nevertheless, these approaches are still widely applied in many pig producing countries where such legal restrictions are not yet in place, although this situation is expected to evolve in the medium term as a One Health framework is progressively implemented worldwide [31]. Finally, other measures such as improved husbandry practices, increasing weaning weight, and upgrading facilities should have been prioritized by pig production companies to cope with the additional challenges during the nursery period posed by the sanitary situation (HPPRRSV strain) and by recent restrictions on the use of antimicrobials and zinc oxide. The implementation of these measures has been uneven in the Spanish pig sector in the study period (Miguel angel Higuera, Anprograpor president, personal communication).

The ADG and FCR have generally improved in the fattening phase between 2010 [22] and 2021 [this study]. Afterwards, this tendency changed and an impairment in both parameters were observed until 2023. The main reasons to explain this evolution between 2021 and 2023 could be the increase in the slaughter weight (5 kilograms from 2021 to 2023) and in the fattening mortality (2% in this period). It has been thoroughly described that an increase in slaughter weight could be directly associated with an increase in average daily gain and a higher feed conversion ratio [16]. Moreover, it has been also described that an increase on the fattening mortality could also affect negatively the feed conversion ratio and average daily gain [16]. Remarkably, when both parameters were adjusted at the same slaughter weight, the tendency for both fattening parameters in the period 2021–2023 was very close to the one observed during the period 2010–2021. Thus, our results suggest that the fattening average daily gain and feed conversion ratio in Spain were expected to increase and decrease by 7 grams/day/year and 0.025 per year, respectively in the period 2010–2024 (considering the adjustment previously commented), corroborating the objectives of pig breeding programs that prioritize improvement in feed conversion ratio as a selection criteria [24, 32] and in line with the leading pig-producing countries in Europe [23].

Mortality rates during nursery and fattening phases are directly linked to reduced profitability in swine production systems [17]. Between 2010 and 2014, mortality steadily declined in Spain [22]. However, this tendency changed in 2015, when the mortality during the rearing period steadily increased until 2021, where a steep slope was observed. (Figs. 4 and 5). This increase can be attributed to a multifactorial effect where the introduction of a high virulent PRRSV strain since 2020 was probably a key player, as suggested by its significant role in swine pathology [4]. On the other hand, the ban of zinc oxide in 2022 could even have complicated the sanitary situation in nurseries with a roll-over effect in the fattening phase. The pig sector is doing a huge effort to decrease mortality in the nursery and fattening period such as active surveillance of PRRSV in semen, improve internal and external biosecurity and increase the number of sow farms working in 4–5 batch production systems. The efficacy of these measures will be monitored in the coming years.

## Cost of production

The total cost per produced kilogram of body weight decreased from 2015 to 2016 and steadily increased from 2017 to 2020. Afterwards, this parameter suffered a tremendous increase, reaching the maximum value in 2022. After that year, both decreased but not returned to the values observed in the previous years (2015–2020). The total cost of pig production can be categorized into TFC, TFIXC, TREPC, and DVCT [22, 23]. In addition, it is important to consider that sow productivity and post-weaning mortality affect all the cost components. The worsening of these factors during the last years, has represented an increase in all the different costs, because the expenses must be inputted between a lower number of animals [23].

In Spain, TFC accounted for approximately 65.4–73.3% of the TCP3 over the past ten years, with considerable annual variation attributable to significant fluctuations in feed prices that has been extreme in the period 2021–2023 (69.0–73.3%) [22, this study]. Notably, this proportion has remained below 65% in other major European pig-producing countries, including Denmark, France, Germany, and the Netherlands, likely reflecting consistently higher feed costs in Spain compared to these countries [23]. This disparity may be explained by the chronic deficiency in domestic cereal production required to supply Spanish feed mills [33] that has been even worse due to the Ukraine war that has produced a shortage on the cereal availability with a huge increase in its price. Production costs for weaned, nursery, and fattening pigs have mirrored trends in feed prices for corresponding production phases over the study period. These costs would likely have shown a continuous decline in the period where feed prices remained stable, due to improvements in technical parameters observed in each phase. Importantly, our findings indicate that FCR holds the greatest economic significance under high feed price conditions, corroborating results reported by other researchers [34].

The second factor more relevant for the total cost is the TFIXC. This parameter has been continuously increasing during the study period. During this timeframe, the labour expenses, construction of facilities, facilities upgrades and input costs such as energy has been steadily increasing in Spain with a steep slope since 2020 [35]. In relation with the DVCT per pig, the pattern observed showed a steadily increase during the whole study period with a steep slope since 2021. This pattern may be explained by the sanitary situation previously discussed and the increase in the cost of drug and vaccines across the study period. Finally, the TREPC per pig, that contains the insemination process and the cost of the gilts, is more complex to be disentangled and analysed. The main reason is because the price of the slaughterhouse sow is considered in this calculus, and it can vary a lot on the different years. For this reason, it is difficult to analyse the tendency of this factor. In any case, the cost of reproduction is the lowest of all the components involved in the pig producing cost [23].

Finally, our results indicate that the number of piglets born alive per litter, kilograms of sow feed per weaned piglet, fattening feed conversion ratio, total feed conversion ratio, and total feed costs are significantly worse in southern Spain than in other pig-producing regions of the country. In contrast, the nursery average daily gain and the preweaning mortality are significantly higher and lower, respectively in southern Spain than in other areas. Many of these differences may be attributable to heat stress affecting sows and fattening pigs in southern Spain during late spring, summer, and early autumn which has been reported to impair production performance and elevate production costs [36, 37]. Accordingly, the Spanish pig industry is investing in improved cooling systems to counter the rising frequency and intensity of heat waves [38].

## Conclusions

Pig production performance has generally improved from 2015 to 2020 and 2015 to 2022 during the piglet production and fattening phase, respectively and worsened afterwards. The improvement reflects significant advancements in breeding programs focused mainly on improving prolificacy and FCR. However, this tendency generally changed in 2020 probably due to the appearance of HP-PRRSV strains in the Spanish pig population that is hampering the production despite the genetic improvement. On the other hand, performance in the nursery phase have continuously worsened since 2015 probably due a multifactorial effect caused by the new regulations in the use of antibiotics, the ban to use zinc oxide since 2022 and the introduction of HP-PRRSV strains since 2020. Finally, the cost of pig production has been significantly changed in the last ten years by the huge volatility observed in the feed cost plus the changes observed in the production performance indicators formerly described. To have reliable and updated data, is key for the sector to improve its efficiency and competitiveness. The publication of this article intends to help producers and researchers, providing useful and reliable information that is scarce in peer-review publications.

## Electronic supplementary material

Below is the link to the electronic supplementary material.

Supplementary material 1

Supplementary material 2

Supplementary material 3

Supplementary material 4

Supplementary material 5

Supplementary material 6

## Data Availability

Data and analysis scripts are available from the authors upon request.
