# Supplementary figures and images for: Pig productive performance parameters and costs in Spain: evolution from 2015 to 2024
Source: Porcine Health Manag. 2026 Mar 5;12:17. doi: 10.1186/s40813-026-00500-w (PMC13072534; doi:10.1186/s40813-026-00500-w)

## Slide 1
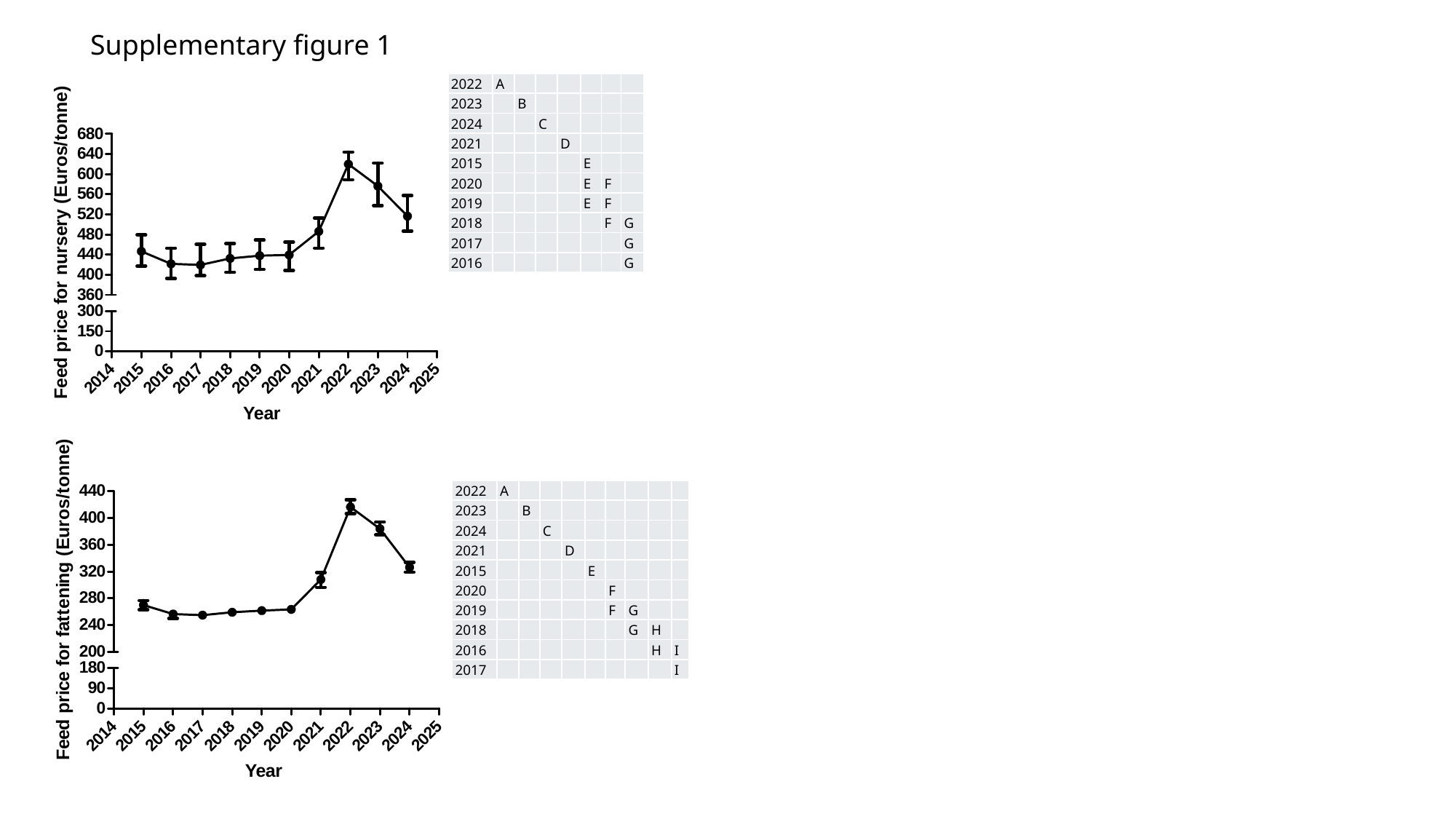

Supplementary figure 1
| 2022 | A | | | | | | |
| --- | --- | --- | --- | --- | --- | --- | --- |
| 2023 | | B | | | | | |
| 2024 | | | C | | | | |
| 2021 | | | | D | | | |
| 2015 | | | | | E | | |
| 2020 | | | | | E | F | |
| 2019 | | | | | E | F | |
| 2018 | | | | | | F | G |
| 2017 | | | | | | | G |
| 2016 | | | | | | | G |
| 2022 | A | | | | | | | | |
| --- | --- | --- | --- | --- | --- | --- | --- | --- | --- |
| 2023 | | B | | | | | | | |
| 2024 | | | C | | | | | | |
| 2021 | | | | D | | | | | |
| 2015 | | | | | E | | | | |
| 2020 | | | | | | F | | | |
| 2019 | | | | | | F | G | | |
| 2018 | | | | | | | G | H | |
| 2016 | | | | | | | | H | I |
| 2017 | | | | | | | | | I |

Supplement: Supplementary file 1 — Supplementary material 1 [file 40813_2026_500_MOESM1_ESM.pptx]
