# Supplementary material for: Pig productive performance parameters and costs in Spain: evolution from 2015 to 2024
Source: Porcine Health Manag. 2026 Mar 5;12:17. doi: 10.1186/s40813-026-00500-w (PMC13072534; doi:10.1186/s40813-026-00500-w)
Supplement: Supplementary file 2 — Supplementary material 2 [file 40813_2026_500_MOESM2_ESM.docx]

Supplementary table 1.- Descriptive statistics (mean and interquartile range) and values estimated with the generalized mixed linear model for all the variables of the piglet production phase.

**Number of piglets born alive per litter**

| Variable | Descriptive statistics | | Values estimated with the generalized mixed linear model | | |
| --- | --- | --- | --- | --- | --- |
| Year | Median | Interquartile range | Least square means | Standard error mean | 95% confidence interval |
| 2015 | 12.9 | 12.1-13.3 | 12.6 | 0.11 | 12.4-12.9 |
| 2016 | 13.1 | 12.5-14 | 13 | 0.12 | 12.8-13.2 |
| 2017 | 13.3 | 12.6-14.4 | 13.3 | 0.11 | 13.1-13.5 |
| 2018 | 13.5 | 12.9-14.6 | 13.6 | 0.11 | 13.4-13.8 |
| 2019 | 13.6 | 12.7-15.2 | 13.8 | 0.11 | 13.6-14 |
| 2020 | 14.1 | 13.1-15.6 | 14 | 0.11 | 13.8-14.3 |
| 2021 | 13.9 | 13.2-15.5 | 14.1 | 0.11 | 13.9-14.3 |
| 2022 | 13.9 | 13.3-15.2 | 14 | 0.11 | 13.8-14.3 |
| 2023 | 14.1 | 13.3-15.4 | 14.1 | 0.11 | 13.9-14.3 |
| 2024 | 14.3 | 13.6-15.5 | 14.3 | 0.11 | 14.1-14.5 |

| Variable | Descriptive statistics | | Values estimated with the generalized mixed linear model | | |
| --- | --- | --- | --- | --- | --- |
| Geographical area | Median | Interquartile range | Least square means | Standard error mean | 95% confidence interval |
| East | 13.8 | 12.9-15.2 | 13.1 | 0.11 | 12.8-13.3 |
| North | 13.9 | 13.4-14.7 | 12.7 | 0.21 | 12.3-13.1 |
| South | 13.1 | 12.5-13.7 | 12.2 | 0.20 | 11.8-12.6 |

**Preweaning mortality (%)**

| Variable | Descriptive statistics | | Values estimated with the generalized mixed linear model | | |
| --- | --- | --- | --- | --- | --- |
| Year | Median | Interquartile range | Least square means | Standard error mean | 95% confidence interval |
| 2015 | 13.1 | 10.5-15.4 | 12.3 | 0.39 | 11.5-13 |
| 2016 | 13.5 | 11.2-15.5 | 12.7 | 0.38 | 12-13.5 |
| 2017 | 13.7 | 11.5-15.9 | 13.2 | 0.37 | 12.4-13.9 |
| 2018 | 13.6 | 11.4-16 | 13.3 | 0.37 | 12.6-14.1 |
| 2019 | 13.8 | 11.3-16 | 13.7 | 0.38 | 13-14.4 |
| 2020 | 13.9 | 11.6-16 | 13.9 | 0.38 | 13.2-14.7 |
| 2021 | 14 | 11.5-17.3 | 14.1 | 0.38 | 13.3-14.8 |
| 2022 | 14.5 | 11.9-18.1 | 14.6 | 0.38 | 13.9-15.3 |
| 2023 | 15.4 | 12.2-19 | 15.1 | 0.38 | 14.3-15.8 |
| 2024 | 15.9 | 12.1-19.4 | 15.1 | 0.37 | 14.4-15.8 |

| Variable | Descriptive statistics | | Values estimated with the generalized mixed linear model | | |
| --- | --- | --- | --- | --- | --- |
| Geographical area | Median | Interquartile range | Least square means | Standard error mean | 95% confidence interval |
| East | 14.6 | 12-17.4 | 13.6 | 0.4 | 12.8-14.4 |
| North | 14 | 12.4-16.2 | 12.2 | 0.7 | 11-13.5 |
| South | 11.4 | 9.3-13.6 | 10.9 | 0.6 | 9.7-12.2 |

**Number of piglets weaned per sow per litter**

| Variable | Descriptive statistics | | Values estimated with the generalized mixed linear model | | |
| --- | --- | --- | --- | --- | --- |
| Year | Median | Interquartile range | Least square means | Standard error mean | 95% confidence interval |
| 2015 | 11.1 | 10.7-11.6 | 11.1 | 0.09 | 10.9-11.3 |
| 2016 | 11.4 | 10.9-12.1 | 11.3 | 0.09 | 11.2-11.5 |
| 2017 | 11.5 | 11-12.3 | 11.6 | 0.08 | 11.4-11.7 |
| 2018 | 11.7 | 11.2-12.6 | 11.8 | 0.08 | 11.6-11.9 |
| 2019 | 11.7 | 11.3-12.8 | 11.9 | 0.08 | 11.7-12 |
| 2020 | 12 | 11.4-13 | 12.1 | 0.08 | 11.9-12.2 |
| 2021 | 12 | 11.6-13 | 12.1 | 0.08 | 11.9-12.2 |
| 2022 | 11.9 | 11.4-12.6 | 12 | 0.08 | 11.8-12.1 |
| 2023 | 11.9 | 11.5-12.6 | 12 | 0.08 | 11.8-12.1 |
| 2024 | 12.1 | 11.7-12.7 | 12.1 | 0.08 | 11.9-12.3 |

Geographical area was not significant in the generalized mixed linear model

**Number of piglets weaned per sow per year**

| Variable | Descriptive statistics | | Values estimated with the generalized mixed linear model | | |
| --- | --- | --- | --- | --- | --- |
| Year | Median | Interquartile range | Least square means | Standard error mean | 95% confidence interval |
| 2015 | 26 | 24.7-27.5 | 26 | 0.24 | 25.6-26.5 |
| 2016 | 26.7 | 25.3-28.5 | 26.6 | 0.23 | 26.1-27.1 |
| 2017 | 26.8 | 25.0-28.4 | 26.8 | 0.23 | 26.3-27.2 |
| 2018 | 27.4 | 25.7-29 | 27.3 | 0.23 | 26.8-27.7 |
| 2019 | 27.1 | 25.5-29.8 | 27.3 | 0.23 | 26.8-27.7 |
| 2020 | 27.8 | 26-29.7 | 27.8 | 0.23 | 27.3-28.2 |
| 2021 | 27.7 | 26-29.3 | 27.5 | 0.23 | 27-27.9 |
| 2022 | 27.2 | 25.7-28.6 | 26.9 | 0.23 | 26.5-27.4 |
| 2023 | 26.9 | 24.5-28.5 | 26.5 | 0.23 | 26-26.9 |
| 2024 | 27.1 | 24.9-28.5 | 26.7 | 0.23 | 26.2-27.1 |

Geographical area was not significant in the generalized mixed linear model

**Number of cycles per sow per year**

| Variable | Descriptive statistics | | Values estimated with the generalized mixed linear model | | |
| --- | --- | --- | --- | --- | --- |
| Year | Median | Interquartile range | Least square means | Standard error mean | 95% confidence interval |
| 2015 | 2.34 | 2.29-2.39 | 2.34 | 0.009 | 2.33-2.36 |
| 2016 | 2.34 | 2.29-2.38 | 2.35 | 0.009 | 2.33-2.36 |
| 2017 | 2.31 | 2.26-2.36 | 2.31 | 0.009 | 2.30-2.33 |
| 2018 | 2.31 | 2.26-2.36 | 2.32 | 0.009 | 2.30-2.33 |
| 2019 | 2.29 | 2.24-2.35 | 2.30 | 0.009 | 2.28-2.32 |
| 2020 | 2.30 | 2.23-2.35 | 2.30 | 0.009 | 2.28-2.32 |
| 2021 | 2.28 | 2.24-2.33 | 2.28 | 0.009 | 2.26-2.29 |
| 2022 | 2.26 | 2.19-2.31 | 2.25 | 0.009 | 2.23-2.27 |
| 2023 | 2.22 | 2.16-2.29 | 2.21 | 0.009 | 2.20-2.23 |
| 2024 | 2.21 | 2.14-2.27 | 2.21 | 0.009 | 2.19-2.22 |

Geographical area was not significant in the generalized mixed linear model

**Sow mortality (%)**

| Variable | Descriptive statistics | | Values estimated with the generalized mixed linear model | | |
| --- | --- | --- | --- | --- | --- |
| Year | Median | Interquartile range | Least square means | Standard error mean | 95% confidence interval |
| 2015 | 8 | 5.8-10.3 | 7.6 | 0.4 | 6.8-8.5 |
| 2016 | 8 | 5.9-10.4 | 7.7 | 0.4 | 6.9-8.6 |
| 2017 | 8.7 | 6.9-10.7 | 8.5 | 0.4 | 7.6-9.3 |
| 2018 | 9.5 | 7.2-11.8 | 9.1 | 0.4 | 8.3-9.9 |
| 2019 | 10.2 | 8.1-12.7 | 10 | 0.4 | 9.2-10.8 |
| 2020 | 11.1 | 8.5-14.8 | 10.7 | 0.4 | 9.9-11.6 |
| 2021 | 12.2 | 9.6-15.5 | 11.7 | 0.4 | 10.9-12.5 |
| 2022 | 13.3 | 9.8-17.9 | 13.2 | 0.4 | 12.4-14.1 |
| 2023 | 15.1 | 11.3-18.3 | 14.4 | 0.4 | 13.6-15.2 |
| 2024 | 14.3 | 10.9-17.7 | 13.9 | 0.4 | 13.1-14.7 |

| Variable | Descriptive statistics | | Values estimated with the generalized mixed linear model | | |
| --- | --- | --- | --- | --- | --- |
| Geographical area | Median | Interquartile range | Least square means | Standard error mean | 95% confidence interval |
| East | 11.2 | 8-15.2 | 8.9 | 0.4 | 8-9.8 |
| North | 10 | 7.4-13.9 | 7.1 | 0.7 | 5.7-8.5 |
| South | 9.6 | 7.3-12.1 | 6.9 | 0.7 | 5.4-8.2 |

**Feed price per sow (Euros/tonne)**

| Variable | Descriptive statistics | | Values estimated with the generalized mixed linear model | | |
| --- | --- | --- | --- | --- | --- |
| Year | Median | Interquartile range | Least square means | Standard error mean | 95% confidence interval |
| 2015 | 246.9 | 237.4-255.3 | 245.4 | 1.5 | 242.4-248.4 |
| 2016 | 232 | 222.8-241.8 | 231.4 | 1.5 | 228.5-234.4 |
| 2017 | 233.1 | 223.1-243.5 | 231.8 | 1.5 | 228.9-234.7 |
| 2018 | 242 | 235.1-254 | 243.1 | 1.5 | 240.2-246 |
| 2019 | 248 | 237.4-258.9 | 247.8 | 1.5 | 244.8-250.7 |
| 2020 | 249.4 | 241.5-260.1 | 250.5 | 1.5 | 247.5-253.3 |
| 2021 | 294 | 283.2-306.3 | 294.4 | 1.5 | 291.5-297.2 |
| 2022 | 404.6 | 392.7-416 | 403.7 | 1.5 | 400.8-406.6 |
| 2023 | 367.8 | 356.4-386.8 | 370.3 | 1.5 | 367.4-373.3 |
| 2024 | 303.8 | 293.6-317.2 | 305.8 | 1.5 | 302.9-308.7 |

Geographical area was not significant in the generalized mixed linear model

**Kilograms of sow feed per weaned piglet**

| Variable | Descriptive statistics | | Values estimated with the generalized mixed linear model | | |
| --- | --- | --- | --- | --- | --- |
| Year | Median | Interquartile range | Least square means | Standard error mean | 95% confidence interval |
| 2015 | 42.4 | 40.3-45.4 | 43.5 | 0.4 | 42.8-44.3 |
| 2016 | 42 | 38.9-44.3 | 42.6 | 0.4 | 41.8-43.3 |
| 2017 | 41.6 | 38.6-44.1 | 42.2 | 0.4 | 41.4-42.9 |
| 2018 | 41.7 | 39-44.3 | 42.4 | 0.4 | 41.7-43.2 |
| 2019 | 41.6 | 39.1-44.6 | 42.8 | 0.4 | 42.1-43.6 |
| 2020 | 41.4 | 39.7-45.2 | 42.7 | 0.4 | 41.9-43.4 |
| 2021 | 42.7 | 39.7-44.7 | 43.1 | 0.4 | 42.3-43.8 |
| 2022 | 43.3 | 41.3-45.2 | 43.8 | 0.4 | 43.1-44.5 |
| 2023 | 43.7 | 41.1-47.7 | 45.1 | 0.4 | 44.4-45.9 |
| 2024 | 44.2 | 41.8-47.2 | 45.2 | 0.4 | 44.4-45.9 |

| Variable | Descriptive statistics | | Values estimated with the generalized mixed linear model | | |
| --- | --- | --- | --- | --- | --- |
| Geographical area | Median | Interquartile range | Least square means | Standard error mean | 95% confidence interval |
| East | 42.3 | 39.5-45.1 | 42.9 | 0.4 | 42.1-43.6 |
| North | 41.5 | 40.1-43.7 | 42.2 | 0.6 | 40.9-43.5 |
| South | 44.6 | 42.6-46.8 | 45.4 | 0.6 | 44.2-46.7 |

**Total kilograms of sow feed per year**

| Variable | Descriptive statistics | | Values estimated with the generalized mixed linear model | | |
| --- | --- | --- | --- | --- | --- |
| Year | Median | Interquartile range | Least square means | Standard error mean | 95% confidence interval |
| 2015 | 1124.3 | 1079.2-1157 | 1128.6 | 6.4 | 1116.1-1141.1 |
| 2016 | 1122.9 | 1074.2-1159.9 | 1125.6 | 6.3 | 1113.2-1137.9 |
| 2017 | 1113.3 | 1075-1159.8 | 1121.5 | 6.1 | 1109.4-1133.6 |
| 2018 | 1138.9 | 1101-1172.2 | 1146.8 | 6.1 | 1134.7-1158.8 |
| 2019 | 1150.8 | 1102-1200 | 1156.9 | 6.2 | 1144.8-1169.1 |
| 2020 | 1157.9 | 1132.9-1209 | 1173.5 | 6.2 | 1161.3-1185.8 |
| 2021 | 1181.1 | 1120.3-1224.9- | 1177.8 | 6.2 | 1165.7-1190 |
| 2022 | 1169.1 | 1122.1-1208.3 | 1172.8 | 6.2 | 1160.6-1184.9 |
| 2023 | 1176.8 | 1138.6-1225.3 | 1185 | 6.2 | 1172.8-1197.2 |
| 2024 | 1194.4 | 1142.1-1245.4 | 1198.6 | 6.1 | 1186.6-1210.7 |

| Variable | Descriptive statistics | | Values estimated with the generalized mixed linear model | | |
| --- | --- | --- | --- | --- | --- |
| Geographical area | Median | Interquartile range | Least square means | Standard error mean | 95% confidence interval |
| East | 1148.5 | 1100.9-1189.9 | 1118.2 | 6.4 | 1105.7-1130.7 |
| North | 1155.1 | 1131.1-1207.9 | 1115.5 | 10.3 | 1095.2-1135.7 |
| South | 1176.9 | 1125.6-1229.7 | 1152 | 10.3 | 1131.8-1172.2 |

**Cost per weaned piglet (Euros)**

| Variable | Descriptive statistics | | Values estimated with the generalized mixed linear model | | |
| --- | --- | --- | --- | --- | --- |
| Year | Median | Interquartile range | Least square means | Standard error mean | 95% confidence interval |
| 2015 | 26.6 | 24.2-28.2 | 26.4 | 0.3 | 25.8-27 |
| 2016 | 24.9 | 23.2-26.7 | 24.9 | 0.3 | 24.3-25.5 |
| 2017 | 25.3 | 23.1-26.6 | 25 | 0.3 | 24.4-25.6 |
| 2018 | 26.5 | 24.9-27.8 | 26.4 | 0.3 | 25.8-27 |
| 2019 | 27 | 25.3-28.8 | 27.2 | 0.3 | 26.6-27.7 |
| 2020 | 28.1 | 25.9-30.1 | 28 | 0.3 | 27.4-28.6 |
| 2021 | 31.4 | 28.8-33 | 31 | 0.3 | 30.4-31.6 |
| 2022 | 37.7 | 34.9-39.8 | 37.8 | 0.3 | 37.2-38.4 |
| 2023 | 37.2 | 34.6-40.6 | 37.3 | 0.3 | 36.7-37.9 |
| 2024 | 35.6 | 33.1-39 | 36 | 0.3 | 35.5-36.6 |

Geographical area was not significant in the generalized mixed linear model
