# Supplementary material for: Pig productive performance parameters and costs in Spain: evolution from 2015 to 2024
Source: Porcine Health Manag. 2026 Mar 5;12:17. doi: 10.1186/s40813-026-00500-w (PMC13072534; doi:10.1186/s40813-026-00500-w)
Supplement: Supplementary file 3 — Supplementary material 3 [file 40813_2026_500_MOESM3_ESM.docx]

Supplementary table 2.- Descriptive statistics (mean and interquartile range) and values estimated with the generalized mixed linear model for all the variables of the nursery phase (from weaning to 19 Kg of body weight).

**Nursery average daily gain (g/day)**

| Variable | Descriptive statistics | | Values estimated with the generalized mixed linear model | | |
| --- | --- | --- | --- | --- | --- |
| Year | Median | Interquartile range | Least square means | Standard error mean | 95% confidence interval |
| 2015 | 288.3 | 270.8-314.2 | 300.3 | 3.8 | 292.8-307.7 |
| 2016 | 283.6 | 263.7-302.3 | 294.9 | 3.7 | 287.6-302.2 |
| 2017 | 288.7 | 266.2-312.5 | 300.4 | 3.6 | 293.3-307.5 |
| 2018 | 287.2 | 265.4-308.3 | 292.5 | 3.6 | 285.5-299.5 |
| 2019 | 271.7 | 249.3-298.7 | 281.3 | 3.6 | 274.3-288.3 |
| 2020 | 278.9 | 252.3-306.2 | 290.2 | 3.6 | 283.1-297.2 |
| 2021 | 280 | 252-308.4 | 288.6 | 3.6 | 281.7-295.6 |
| 2022 | 262.9 | 226.7-289.2 | 267.7 | 3.6 | 260.6-274.7 |
| 2023 | 258.9 | 230.3-287.2 | 264.3 | 3.6 | 257.2-271.4 |
| 2024 | 262.7 | 231.8-292.8 | 268.2 | 3.6 | 261.2-275.3 |

| Variable | Descriptive statistics | | Values estimated with the generalized mixed linear model | | |
| --- | --- | --- | --- | --- | --- |
| Geographical area | Median | Interquartile range | Least square means | Standard error mean | 95% confidence interval |
| East | 270.6 | 244.4-292 | 283.9 | 3.8 | 276.5-291.3 |
| North | 291.2 | 261.7-314.9 | 304.8 | 6 | 293-316.6 |
| South | 294.2 | 277.7-316.6 | 312.1 | 5.7 | 300.8-323.4 |

**Nursery feed conversion ratio**

| Variable | Descriptive statistics | | Values estimated with the generalized mixed linear model | | |
| --- | --- | --- | --- | --- | --- |
| Year | Median | Interquartile range | Least square means | Standard error mean | 95% confidence interval |
| 2015 | 1.64 | 1.54-1.71 | 1.64 | 0.014 | 1.62-1.67 |
| 2016 | 1.62 | 1.55-1.69 | 1.64 | 0.013 | 1.61-1.66 |
| 2017 | 1.59 | 1.54-1.66 | 1.61 | 0.013 | 1.59-1.64 |
| 2018 | 1.61 | 1.54-1.69 | 1.64 | 0.013 | 1.61-1.66 |
| 2019 | 1.66 | 1.56-1.72 | 1.66 | 0.013 | 1.64-1.69 |
| 2020 | 1.64 | 1.57-1.71 | 1.65 | 0.013 | 1.62-1.67 |
| 2021 | 1.65 | 1.55-1.74 | 1.66 | 0.013 | 1.63-1.68 |
| 2022 | 1.67 | 1.55-1.76 | 1.67 | 0.013 | 1.65-1.70 |
| 2023 | 1.67 | 1.58-1.78 | 1.7 | 0.013 | 1.67-1.73 |
| 2024 | 1.69 | 1.59-1.80 | 1.72 | 0.013 | 1.69-1.74 |

| Variable | Descriptive statistics | | Values estimated with the generalized mixed linear model | | |
| --- | --- | --- | --- | --- | --- |
| Geographical area | Median | Interquartile range | Least square means | Standard error mean | 95% confidence interval |
| East | 1.62 | 1.55-1.72 | 1.63 | 0.013 | 1.61-1.66 |
| North | 1.62 | 1.54-1.69 | 1.61 | 0.021 | 1.56-1.65 |
| South | 1.69 | 1.61-1.79 | 1.69 | 0.021 | 1.65-1.73 |

**Nursery mortality (%)**

| Variable | Descriptive statistics | | Values estimated with the generalized mixed linear model | | |
| --- | --- | --- | --- | --- | --- |
| Year | Median | Interquartile range | Least square means | Standard error mean | 95% confidence interval |
| 2015 | 3.3 | 2.4-4.3 | 3.4 | 0.27 | 2.8-3.9 |
| 2016 | 3.6 | 2.7-4.9 | 3.7 | 0.26 | 3.2-4.2 |
| 2017 | 3.7 | 2.9-4.8 | 3.5 | 0.25 | 3-4 |
| 2018 | 4.8 | 3.4-5.9 | 4.6 | 0.25 | 4.1-5.1 |
| 2019 | 5.1 | 4-6.6 | 5.2 | 0.26 | 4.7-5.7 |
| 2020 | 4.4 | 3.6-6 | 4.5 | 0.26 | 3.9-5 |
| 2021 | 5.2 | 4-6.5 | 5.1 | 0.25 | 4.6-5.6 |
| 2022 | 7.4 | 5.3-9.6 | 7.5 | 0.25 | 7-8 |
| 2023 | 7.6 | 5.1-10.6 | 8.1 | 0.26 | 7.5-8.6 |
| 2024 | 7.1 | 4.6-9.4 | 7.4 | 0.26 | 6.9-7.9 |

| Variable | Descriptive statistics | | Values estimated with the generalized mixed linear model | | |
| --- | --- | --- | --- | --- | --- |
| Geographical area | Median | Interquartile range | Least square means | Standard error mean | 95% confidence interval |
| East | 4.9 | 3.4-7.2 | 3.9 | 0.26 | 3.4-4.4 |
| North | 4.1 | 3.2-6 | 2.6 | 0.41 | 1.8-3.4 |
| South | 5.1 | 3.8-7.2 | 3.6 | 0.40 | 2.8-3.4 |

**Feed price for nursery (Euros/tonne)**

| Variable | Descriptive statistics | | Values estimated with the generalized mixed linear model | | |
| --- | --- | --- | --- | --- | --- |
| Year | Median | Interquartile range | Least square means | Standard error mean | 95% confidence interval |
| 2015 | 446.5 | 417.5-479.6 | 444.9 | 4.5 | 435.9-453.8 |
| 2016 | 421.7 | 392.7-452.8 | 422.1 | 4.5 | 413.2-430.9 |
| 2017 | 419.6 | 398.4-460.9 | 423.6 | 4.4 | 414.9-432.2 |
| 2018 | 432.6 | 405-462.3 | 431.2 | 4.4 | 422.5-439.9 |
| 2019 | 437.9 | 410.7-469.2 | 439.2 | 4.4 | 430.5-447.9 |
| 2020 | 439.3 | 408.9-465.4 | 441.4 | 4.4 | 432.7-450.1 |
| 2021 | 486.2 | 453.1-512.8 | 486.4 | 4.4 | 477.7-495.1 |
| 2022 | 620.1 | 588.9-644.23 | 616.9 | 4.4 | 608.2-625.6 |
| 2023 | 576.3 | 537.8-622.13 | 585.4 | 4.5 | 576.7-594.2 |
| 2024 | 516.5 | 487-557.8 | 529.2 | 4.4 | 520.4-537.9 |

Geographical area was not significant in the generalized mixed linear model

**Cost per nursery piglet (Euros)**

| Variable | Descriptive statistics | | Values estimated with the generalized mixed linear model | | |
| --- | --- | --- | --- | --- | --- |
| Year | Median | Interquartile range | Least square means | Standard error mean | 95% confidence interval |
| 2015 | 40.6 | 37.2-42.6 | 40 | 0.5 | 39-40.9 |
| 2016 | 38 | 35.9-40.2 | 37.5 | 0.5 | 36.6-38.4 |
| 2017 | 40 | 37.4-42.2 | 39.3 | 0.5 | 38.4-40.2 |
| 2018 | 43.4 | 40.5-45.6 | 42.8 | 0.5 | 41.9-43.7 |
| 2019 | 43.1 | 40.6-45.8 | 42.6 | 0.5 | 41.7-43.5 |
| 2020 | 44.5 | 41.-46.6 | 43.7 | 0.5 | 42.8-44.6 |
| 2021 | 48.1 | 45.6-51.7 | 48.5 | 0.5 | 47.6-49.4 |
| 2022 | 59.6 | 55.4-62.1 | 58.9 | 0.5 | 58-59.8 |
| 2023 | 59.2 | 53.8-63.4 | 58.7 | 0.5 | 57.8-59.6 |
| 2024 | 56.7 | 52.3-61 | 56.5 | 0.5 | 55.6-57.4 |

| Variable | Descriptive statistics | | Values estimated with the generalized mixed linear model | | |
| --- | --- | --- | --- | --- | --- |
| Geographical area | Median | Interquartile range | Least square means | Standard error mean | 95% confidence interval |
| East | 45.4 | 40.5-54.3 | 41.2 | 0.5 | 40.3-42.1 |
| North | 44.4 | 40.5-52.8 | 38.9 | 0.8 | 37.4-40.5 |
| South | 45 | 40.5-53.6 | 39.7 | 0.8 | 38.3-41.2 |
