# Supplementary material for: Pig productive performance parameters and costs in Spain: evolution from 2015 to 2024
Source: Porcine Health Manag. 2026 Mar 5;12:17. doi: 10.1186/s40813-026-00500-w (PMC13072534; doi:10.1186/s40813-026-00500-w)
Supplement: Supplementary file 4 — Supplementary material 4 [file 40813_2026_500_MOESM4_ESM.docx]

Supplementary table 3.- Descriptive statistics (mean and interquartile range) and values estimated with the generalized mixed linear model for all the variables of the fattening phase (from 19 to final Kg of body weight depending on the year-see material and method section).

**Fattening average daily gain (gr/day)**

| Variable | Descriptive statistics | | Values estimated with the generalized mixed linear model | | |
| --- | --- | --- | --- | --- | --- |
| Year | Median | Interquartile range | Least square means | Standard error mean | 95% confidence interval |
| 2015 | 663.8 | 637.7-703.6 | 671.6 | 6.3 | 659.2-684 |
| 2016 | 673.6 | 644.1-708.7 | 684.5 | 6.1 | 672.4-696.6 |
| 2017 | 677.1 | 656.9-701.9 | 680.7 | 6.2 | 668.4-692.9 |
| 2018 | 688.9 | 669.8-725.7 | 696.7 | 6.1 | 684.7-708.7 |
| 2019 | 693 | 669-727.5 | 700 | 6.3 | 687.7-712.4 |
| 2020 | 713.6 | 687.2-739.4 | 722.2 | 6.1 | 710.1-734.2 |
| 2021 | 720 | 678.9-747.8 | 724.7 | 6.1 | 712.6-736.7 |
| 2022 | 709 | 662.8-740.3 | 709.7 | 5.8 | 698.4-721 |
| 2023 | 708.9 | 678.3-749.3 | 711.9 | 5.8 | 700.5-723.3 |
| 2024 | 727.2 | 689.8-750.3 | 725.7 | 5.8 | 714.3-737.1 |

| Variable | Descriptive statistics | | Values estimated with the generalized mixed linear model | | |
| --- | --- | --- | --- | --- | --- |
| Geographical area | Median | Interquartile range | Least square means | Standard error mean | 95% confidence interval |
| East | 699.7 | 671.3-728.8 | 663.7 | 6.4 | 651-676.3 |
| North | 740.9 | 689.7-779.1 | 696.1 | 10.8 | 674.9-717.3 |
| South | 677.1 | 650.5-717.1 | 654.9 | 9.6 | 636.1-673.8 |

**Fattening average daily gain (gr/day) adjusted (standardized to 113 kg across the study period- see material and method section)**

| Variable | Descriptive statistics | | Values estimated with the generalized mixed linear model | | |
| --- | --- | --- | --- | --- | --- |
| Year | Median | Interquartile range | Least square means | Standard error mean | 95% confidence interval |
| 2015 | 681 | 655-721 | 689 | 6.3 | 676.5-701.4 |
| 2016 | 688 | 658.8-723.3 | 698.9 | 6.1 | 686.9-711 |
| 2017 | 688.5 | 668.3-713.3 | 691.9 | 6.2 | 679.6-704.1 |
| 2018 | 697 | 678-734 | 705.2 | 6.1 | 693.2-717.2 |
| 2019 | 699 | 675-733 | 705.7 | 6.3 | 693.3-718.1 |
| 2020 | 713.5 | 687.5-739.3 | 722.1 | 6.1 | 710.1-734.2 |
| 2021 | 717 | 675.8-745 | 721.8 | 6.1 | 709.8-773.9 |
| 2022 | 709 | 663-740 | 709.7 | 5.8 | 698.3-721 |
| 2023 | 698 | 667.5-738.3 | 701.2 | 5.8 | 689.7-712.6 |
| 2024 | 711.5 | 674-734 | 709.7 | 5.8 | 698.2-721.1 |

| Variable | Descriptive statistics | | Values estimated with the generalized mixed linear model | | |
| --- | --- | --- | --- | --- | --- |
| Geographical area | Median | Interquartile range | Least square means | Standard error mean | 95% confidence interval |
| East | 700 | 671-730 | 681 | 6.4 | 668.3-693.7 |
| North | 742 | 695-772 | 713.5 | 10.8 | 692.3-734.7 |
| South | 682 | 656-718 | 672.3 | 9.6 | 653.5-691.2 |

**Fattening feed conversion ratio**

| Variable | Descriptive statistics | | Values estimated with the generalized mixed linear model | | |
| --- | --- | --- | --- | --- | --- |
| Year | Median | Interquartile range | Least square means | Standard error mean | 95% confidence interval |
| 2015 | 2.54 | 2.48-2.62 | 2.57 | 0.012 | 2.55-2.59 |
| 2016 | 2.50 | 2.42-2.57 | 2.54 | 0.012 | 2.51-2.56 |
| 2017 | 2.49 | 2.43-2.55 | 2.52 | 0.012 | 2.50-2.55 |
| 2018 | 2.49 | 2.43-2.57 | 2.51 | 0.012 | 2.49-2.54 |
| 2019 | 2.46 | 2.37-2.55 | 2.50 | 0.012 | 2.47-2.52 |
| 2020 | 2.46 | 2.36-2.55 | 2.48 | 0.012 | 2.46-2.50 |
| 2021 | 2.45 | 2.35-2.54 | 2.47 | 0.012 | 2.45-2.49 |
| 2022 | 2.44 | 2.35-2.56 | 2.49 | 0.012 | 2.47-2.51 |
| 2023 | 2.48 | 2.38-2.56 | 2.50 | 0.012 | 2.48-2.52 |
| 2024 | 2.46 | 2.37-2.52 | 2.50 | 0.012 | 2.47-2.52 |

| Variable | Descriptive statistics | | Values estimated with the generalized mixed linear model | | |
| --- | --- | --- | --- | --- | --- |
| Geographical area | Median | Interquartile range | Least square means | Standard error mean | 95% confidence interval |
| East | 2.46 | 2.37-2.53 | 2.53 | 0.012 | 2.51-2.56 |
| North | 2.50 | 2.43-2.56 | 2.55 | 0.022 | 2.51-2.59 |
| South | 2.56 | 2.49-2.63 | 2.62 | 0.019 | 2.59-2.66 |

**Fattening feed conversion ratio adjusted** **(standardized to 113 kg across the study period- see material and method section)**

| Variable | Descriptive statistics | | Values estimated with the generalized mixed linear model | | |
| --- | --- | --- | --- | --- | --- |
| Year | Median | Interquartile range | Least square means | Standard error mean | 95% confidence interval |
| 2015 | 2.59 | 2.52-2.67 | 2.62 | 0.012 | 2.59-2.64 |
| 2016 | 2.54 | 2.46-2.62 | 2.58 | 0.012 | 2.55-2.60 |
| 2017 | 2.52 | 2.46-2.58 | 2.55 | 0.012 | 2.53-2.58 |
| 2018 | 2.52 | 2.45-2.60 | 2.53 | 0.012 | 2.51-2.56 |
| 2019 | 2.47 | 2.39-2.56 | 2.51 | 0.012 | 2.49-2.53 |
| 2020 | 2.46 | 2.37-2.55 | 2.48 | 0.012 | 2.46-2.50 |
| 2021 | 2.44 | 2.34-2.53 | 2.46 | 0.012 | 2.44-2.48 |
| 2022 | 2.45 | 2.35-2.56 | 2.49 | 0.012 | 2.47-2.51 |
| 2023 | 2.44 | 2.35-2.53 | 2.47 | 0.012 | 2.45-2.49 |
| 2024 | 2.42 | 2.33-2.48 | 2.45 | 0.012 | 2.43-2.48 |

| Variable | Descriptive statistics | | Values estimated with the generalized mixed linear model | | |
| --- | --- | --- | --- | --- | --- |
| Geographical area | Median | Interquartile range | Least square means | Standard error mean | 95% confidence interval |
| East | 2.47 | 2.38-2.55 | 2.57 | 0.012 | 2.55-2.60 |
| North | 2.50 | 2.43-2.56 | 2.60 | 0.022 | 2.55-2.64 |
| South | 2.56 | 2.48-2.65 | 2.67 | 0.019 | 2.63-2.71 |

**Fattening mortality (%)**

| Variable | Descriptive statistics | | Values estimated with the generalized mixed linear model | | |
| --- | --- | --- | --- | --- | --- |
| Year | Median | Interquartile range | Least square means | Standard error mean | 95% confidence interval |
| 2015 | 3.6 | 3.1-4.8 | 4 | 0.2 | 3.6-4.4 |
| 2016 | 3.5 | 2.9-4.6 | 3.8 | 0.2 | 3.4-4.1 |
| 2017 | 3.7 | 2.8-4.5 | 3.7 | 0.2 | 3.4-4.1 |
| 2018 | 3.8 | 3-5 | 3.9 | 0.2 | 3.6-4.3 |
| 2019 | 4.1 | 3.2-5.2 | 4.3 | 0.2 | 3.9-4.6 |
| 2020 | 3.8 | 3.2-4.5 | 3.9 | 0.2 | 3.6-4.3 |
| 2021 | 4.1 | 3.2-5 | 4.2 | 0.2 | 3.9-4.6 |
| 2022 | 5.1 | 4-6.4 | 5.4 | 0.2 | 5.1-5.8 |
| 2023 | 5.5 | 4.3-7 | 5.8 | 0.2 | 5.4-6.2 |
| 2024 | 6 | 4.5-7.7 | 6.1 | 0.2 | 5.8-6.5 |

Geographical area was not significant in the generalized mixed linear model

**Feed price for fattening (Euros/tonne)**

| Variable | Descriptive statistics | | Values estimated with the generalized mixed linear model | | |
| --- | --- | --- | --- | --- | --- |
| Year | Median | Interquartile range | Least square means | Standard error mean | 95% confidence interval |
| 2015 | 270.2 | 263-276.8 | 270.3 | 1.4 | 267.5-273.2 |
| 2016 | 256.6 | 250.2-259.4 | 254.9 | 1.4 | 252.2-257.6 |
| 2017 | 255.1 | 250.3-259.4 | 254.2 | 1.4 | 251.5-256.9 |
| 2018 | 259.2 | 255-264 | 259 | 1.4 | 256.3-261.6 |
| 2019 | 261.7 | 255.8-265.9 | 261 | 1.4 | 258.3-263.7 |
| 2020 | 263.8 | 258.4-269.6 | 264.6 | 1.4 | 261.9-267.3 |
| 2021 | 308.2 | 296.7-318.4 | 306.6 | 1.4 | 303.9-309.3 |
| 2022 | 416.2 | 406.3-427.1 | 414.2 | 1.4 | 411.5-416.9 |
| 2023 | 383.9 | 375-393.9 | 385.5 | 1.4 | 382.8-388.3 |
| 2024 | 326.4 | 319.2-334 | 327 | 1.4 | 324.2-329.7 |

Geographical area was not significant in the generalized mixed linear model

**Total cost per pig (Euros)**

| Variable | Descriptive statistics | | Values estimated with the generalized mixed linear model | | |
| --- | --- | --- | --- | --- | --- |
| Year | Median | Interquartile range | Least square means | Standard error mean | 95% confidence interval |
| 2015 | 117.3 | 113.4-121.4 | 115.8 | 0.9 | 114.1-117.6 |
| 2016 | 111.4 | 107.7-114.3 | 109.8 | 0.9 | 108.1-111.5 |
| 2017 | 112.1 | 108.5-114.5 | 112.1 | 0.9 | 110.4-113.8 |
| 2018 | 116.6 | 112.7-120 | 118 | 0.9 | 116.3-119.7 |
| 2019 | 119.4 | 115.2-123 | 119.4 | 0.9 | 117.7-121.1 |
| 2020 | 121.8 | 117-127.7 | 122.8 | 0.9 | 121.1-124.5 |
| 2021 | 136.2 | 132.4-141.8 | 137.8 | 0.9 | 136.1-139.5 |
| 2022 | 174.3 | 166.8-182.1 | 175.3 | 0.9 | 173.6-177 |
| 2023 | 172.3 | 172.3-181.2 | 174.2 | 0.9 | 172.5-175.9 |
| 2024 | 159.2 | 151.8-173.3 | 161.4 | 0.9 | 159.7-163.1 |

Geographical area was not significant in the generalized mixed linear model
