# Supplementary material for: Pig productive performance parameters and costs in Spain: evolution from 2015 to 2024
Source: Porcine Health Manag. 2026 Mar 5;12:17. doi: 10.1186/s40813-026-00500-w (PMC13072534; doi:10.1186/s40813-026-00500-w)
Supplement: Supplementary file 5 — Supplementary material 5 [file 40813_2026_500_MOESM5_ESM.docx]

Supplementary table 4.- Descriptive statistics (mean and interquartile range) and values estimated with the generalized mixed linear model for all the variables of the whole production phase.

**Total feed conversion ratio**

| Variable | Descriptive statistics | | Values estimated with the generalized mixed linear model | | |
| --- | --- | --- | --- | --- | --- |
| Year | Median | Interquartile range | Least square means | Standard error mean | 95% confidence interval |
| 2015 | 2.70 | 2.63-2.79 | 2.72 | 0.012 | 2.7-2.74 |
| 2016 | 2.66 | 2.59-2.73 | 2.69 | 0.012 | 2.67-2.71 |
| 2017 | 2.64 | 2.57-2.71 | 2.67 | 0.012 | 2.64-2.69 |
| 2018 | 2.65 | 2.58-2.73 | 2.66 | 0.012 | 2.64-2.69 |
| 2019 | 2.65 | 2.56-2.73 | 2.66 | 0.012 | 2.64-2.69 |
| 2020 | 2.63 | 2.52-2.73 | 2.64 | 0.012 | 2.62-2.67 |
| 2021 | 2.62 | 2.52-2.72 | 2.64 | 0.012 | 2.61-2.66 |
| 2022 | 2.66 | 2.55-2.76 | 2.68 | 0.012 | 2.66-2.70 |
| 2023 | 2.66 | 2.58-2.77 | 2.69 | 0.012 | 2.67-2.72 |
| 2024 | 2.66 | 2.57-2.74 | 2.69 | 0.012 | 2.67-2.71 |

| Variable | Descriptive statistics | | Values estimated with the generalized mixed linear model | | |
| --- | --- | --- | --- | --- | --- |
| Geographical area | Median | Interquartile range | Least square means | Standard error mean | 95% confidence interval |
| East | 2.63 | 2.55-2.72 | 2.69 | 0.012 | 2.67-2.72 |
| North | 2.65 | 2.58-2.70 | 2.69 | 0.023 | 2.64-2.73 |
| South | 2.73 | 2.67-2.81 | 2.79 | 0.020 | 2.75-2.82 |

**Total feed conversion ratio adjusted (standardized to 113 kg across the study period- see material and method section)**

| Variable | Descriptive statistics | | Values estimated with the generalized mixed linear model | | |
| --- | --- | --- | --- | --- | --- |
| Year | Median | Interquartile range | Least square means | Standard error mean | 95% confidence interval |
| 2015 | 2.73 | 2.65-2.82 | 2.75 | 0.012 | 2.73-2.77 |
| 2016 | 2.69 | 2.61-2.75 | 2.71 | 0.012 | 2.69-2.73 |
| 2017 | 2.66 | 2.58-2.73 | 2.69 | 0.012 | 2.66-2.71 |
| 2018 | 2.66 | 2.60-2.74 | 2.68 | 0.012 | 2.65-2.70 |
| 2019 | 2.67 | 2.57-2.74 | 2.67 | 0.012 | 2.65-2.70 |
| 2020 | 2.63 | 2.52-2.73 | 2.64 | 0.012 | 2.62-2.67 |
| 2021 | 2.62 | 2.52-2.72 | 2.63 | 0.012 | 2.61-2.66 |
| 2022 | 2.66 | 2.55-2.76 | 2.68 | 0.012 | 2.66-2.70 |
| 2023 | 2.65 | 2.56-2.76 | 2.68 | 0.012 | 2.66-2.70 |
| 2024 | 2.64 | 2.55-2.71 | 2.67 | 0.012 | 2.64-2.69 |

| Variable | Descriptive statistics | | Values estimated with the generalized mixed linear model | | |
| --- | --- | --- | --- | --- | --- |
| Geographical area | Median | Interquartile range | Least square means | Standard error mean | 95% confidence interval |
| East | 2.64 | 2.56-2.73 | 2.72 | 0.012 | 2.70-2.75 |
| North | 2.66 | 2.58-2.72 | 2.71 | 0.023 | 2.67-2.76 |
| South | 2.74 | 2.67-2.81 | 2.81 | 0.020 | 2.77-2.86 |

**Total cost per produced Kg of body weight**

| Variable | Descriptive statistics | | Values estimated with the generalized mixed linear model | | |
| --- | --- | --- | --- | --- | --- |
| Year | Median | Interquartile range | Least square means | Standard error mean | 95% confidence interval |
| 2015 | 1.09 | 1.06-1.13 | 1.08 | 0.008 | 1.06-1.09 |
| 2016 | 1.03 | 1.00-1.06 | 1.02 | 0.008 | 1-1.03 |
| 2017 | 1.03 | 1.00-1.05 | 1.03 | 0.008 | 1.01-1.04 |
| 2018 | 1.06 | 1.02-1.09 | 1.07 | 0.008 | 1.06-1.08 |
| 2019 | 1.07 | 1.04-1.11 | 1.07 | 0.008 | 1.06-1.09 |
| 2020 | 1.07 | 1.03-1.13 | 1.08 | 0.008 | 1.07-1.10 |
| 2021 | 1.19 | 1.16-1.24 | 1.21 | 0.008 | 1.19-1.22 |
| 2022 | 1.54 | 1.47-1.61 | 1.55 | 0.008 | 1.53-1.56 |
| 2023 | 1.48 | 1.41-1.55 | 1.49 | 0.008 | 1.48-1.51 |
| 2024 | 1.34 | 1.28-1.40 | 1.36 | 0.008 | 1.34-1.37 |

Geographical area was not significant in the generalized mixed linear model

**Total feed cost per pig (Euros)**

| Variable | Descriptive statistics | | Values estimated with the generalized mixed linear model | | |
| --- | --- | --- | --- | --- | --- |
| Year | Median | Interquartile range | Least square means | Standard error mean | 95% confidence interval |
| 2015 | 81.1 | 78.2-83.3 | 81.6 | 0.6 | 80.5-82.8 |
| 2016 | 75.8 | 73.5-78.2 | 76.6 | 0.6 | 75.4-77.7 |
| 2017 | 75.4 | 73.7-78.3 | 76.6 | 0.6 | 75.5-77.8 |
| 2018 | 78.5 | 75.9-80.9 | 79 | 0.6 | 77.9-80.1 |
| 2019 | 79.9 | 77.1-82.5 | 80.4 | 0.6 | 79.3-81.5 |
| 2020 | 81.9 | 78.5-85.2 | 82.4 | 0.6 | 81.3-83.5 |
| 2021 | 95.3 | 90.5-98.7 | 95.5 | 0.6 | 94.4-96.6 |
| 2022 | 126.5 | 121.6-134 | 129 | 0.6 | 127.9-130.2 |
| 2023 | 122.5 | 118.7-127.1 | 124 | 0.6 | 122.9-125.2 |
| 2024 | 106.4 | 102-111 | 107.7 | 0.6 | 106.6-108.8 |

| Variable | Descriptive statistics | | Values estimated with the generalized mixed linear model | | |
| --- | --- | --- | --- | --- | --- |
| Geographical area | Median | Interquartile range | Least square means | Standard error mean | 95% confidence interval |
| East | 82.5 | 77.2-105.8 | 80.9 | 0.6 | 79.8-82.1 |
| North | 83.5 | 77.2-109.7 | 80 | 1 | 78-82.1 |
| South | 86.5 | 79.7-110.2 | 84 | 0.9 | 82.2-85.8 |

**Total drug and vaccine cost per pig (Euros)**

| Variable | Descriptive statistics | | Values estimated with the generalized mixed linear model | | |
| --- | --- | --- | --- | --- | --- |
| Year | Median | Interquartile range | Least square means | Standard error mean | 95% confidence interval |
| 2015 | 5.3 | 4.2-6.3 | 5.1 | 0.2 | 4.7-5.5 |
| 2016 | 5.3 | 3.9-6.3 | 5 | 0.2 | 4.6-5.3 |
| 2017 | 5.5 | 4.4-6.5 | 5.3 | 0.2 | 4.9-5.6 |
| 2018 | 5.7 | 4.6-6.9 | 5.6 | 0.2 | 5.2-5.9 |
| 2019 | 6 | 5-7.3 | 6 | 0.2 | 5.7-6.4 |
| 2020 | 6.1 | 4.9-7.6 | 6.2 | 0.2 | 5.9-6.6 |
| 2021 | 6.3 | 5.1-7.5 | 6.3 | 0.2 | 5.9-6.6 |
| 2022 | 6.5 | 5.5-7.8 | 6.4 | 0.2 | 6-6.8 |
| 2023 | 7.2 | 6.2-8.9 | 7.2 | 0.2 | 6.8-7.6 |
| 2024 | 7.7 | 6.7-9.1 | 7.7 | 0.2 | 7.3-8.1 |

Geographical area was not significant in the generalized mixed linear model

**Total fixed cost per pig (Euros)**

| Variable | Descriptive statistics | | Values estimated with the generalized mixed linear model | | |
| --- | --- | --- | --- | --- | --- |
| Year | Median | Interquartile range | Least square means | Standard error mean | 95% confidence interval |
| 2015 | 27.5 | 25.1-30 | 27.9 | 0.5 | 27-28.9 |
| 2016 | 26.8 | 25-29 | 27.2 | 0.5 | 26.2-28.2 |
| 2017 | 27.1 | 25.4-29.4 | 27.9 | 0.5 | 26.9-28.8 |
| 2018 | 28 | 25.6-30.8 | 28.3 | 0.5 | 27.4-29.3 |
| 2019 | 29.6 | 27-33.3 | 29.5 | 0.5 | 28.5-30.4 |
| 2020 | 30.3 | 27.3-34 | 30.3 | 0.5 | 29.4-31.3 |
| 2021 | 32.1 | 28.8-35.2 | 31.6 | 0.5 | 30.6-32.5 |
| 2022 | 35.2 | 31.1-38.2 | 34.7 | 0.5 | 33.8-35.7 |
| 2023 | 37.4 | 34.3-42.1 | 37.4 | 0.5 | 36.4-38.3 |
| 2024 | 40.3 | 35.8-43.5 | 39.7 | 0.5 | 38.7-40.7 |

Geographical area was not significant in the generalized mixed linear model

**Total reproduction cost per pig (Euros)**

| Variable | Descriptive statistics | | Values estimated with the generalized mixed linear model | | |
| --- | --- | --- | --- | --- | --- |
| Year | Median | Interquartile range | Least square means | Standard error mean | 95% confidence interval |
| 2015 | 3.2 | 2.8-3.8 | 3.3 | 0.1 | 3-3.6 |
| 2016 | 3 | 2.4-3.6 | 3 | 0.1 | 2.7-3.3 |
| 2017 | 2.7 | 2.2-3.3 | 2.8 | 0.1 | 2.5-3.1 |
| 2018 | 3.2 | 2.6-3.9 | 3.3 | 0.1 | 3-3.6 |
| 2019 | 3.2 | 2.4-3.9 | 3.2 | 0.1 | 2.9-3.5 |
| 2020 | 3.5 | 2.9-4.2 | 3.5 | 0.1 | 3.3-3.8 |
| 2021 | 4.2 | 3.7-4.9 | 4.3 | 0.1 | 4.1-4.6 |
| 2022 | 5.4 | 4.3-6.3 | 5.4 | 0.1 | 5.1-5.7 |
| 2023 | 4.4 | 3.3-5.6 | 4.6 | 0.1 | 4.3-4.8 |
| 2024 | 4.9 | 3.9-6 | 5 | 0.1 | 4.8-5.3 |

Geographical area was not significant in the generalized mixed linear model
