# Supplementary material for: Pig productive performance parameters and costs in Spain: evolution from 2015 to 2024
Source: Porcine Health Manag. 2026 Mar 5;12:17. doi: 10.1186/s40813-026-00500-w (PMC13072534; doi:10.1186/s40813-026-00500-w)
Supplement: Supplementary file 6 — Supplementary material 6 [file 40813_2026_500_MOESM6_ESM.pptx]

## Slide 1
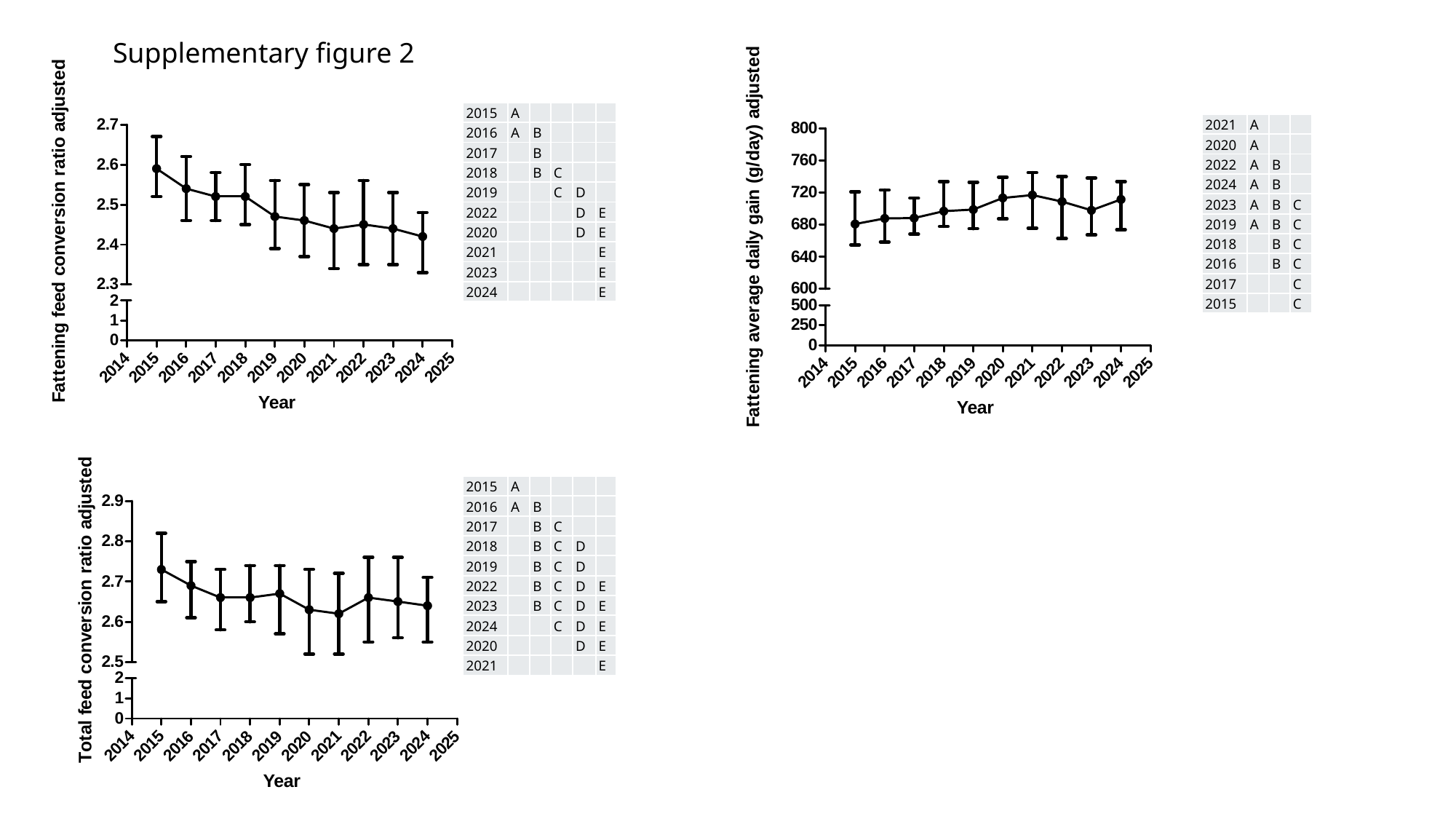

Supplementary figure 2
| 2015 | A | | | | |
| --- | --- | --- | --- | --- | --- |
| 2016 | A | B | | | |
| 2017 | | B | | | |
| 2018 | | B | C | | |
| 2019 | | | C | D | |
| 2022 | | | | D | E |
| 2020 | | | | D | E |
| 2021 | | | | | E |
| 2023 | | | | | E |
| 2024 | | | | | E |
| 2021 | A | | |
| --- | --- | --- | --- |
| 2020 | A | | |
| 2022 | A | B | |
| 2024 | A | B | |
| 2023 | A | B | C |
| 2019 | A | B | C |
| 2018 | | B | C |
| 2016 | | B | C |
| 2017 | | | C |
| 2015 | | | C |
| 2015 | A | | | | |
| --- | --- | --- | --- | --- | --- |
| 2016 | A | B | | | |
| 2017 | | B | C | | |
| 2018 | | B | C | D | |
| 2019 | | B | C | D | |
| 2022 | | B | C | D | E |
| 2023 | | B | C | D | E |
| 2024 | | | C | D | E |
| 2020 | | | | D | E |
| 2021 | | | | | E |
